# The Determinants of Men’s Health Behaviors: A Cross-Sectional Study Among Public Safety Personnel in Kelantan, Malaysia

**DOI:** 10.3390/healthcare13030291

**Published:** 2025-01-31

**Authors:** Muhammad Iqbal Haji Mukhti, Mohd Ismail Ibrahim

**Affiliations:** Department of Community Medicine, School of Medical Sciences, Health Campus, Universiti Sains Malaysia, Kubang Kerian 16150, Kelantan, Malaysia; muhammadiqbal@student.usm.my

**Keywords:** men’s health behaviors, public safety personnel, Andersen’s behavioral model, healthcare access

## Abstract

Background: Men’s health behaviors influence health outcomes but remain understudied in high-risk occupational groups. This study examines determinants of health behaviors among public safety personnel using Andersen’s Behavioral Model. Methods: A cross-sectional study was conducted among 257 male public safety personnel in Kelantan, Malaysia. Participants were selected through simple random sampling. Data were collected using proforma and the validated Malay Health Behavior Inventory-Short Form (MHBI-SF). Multiple logistic regression identified factors associated with poor health behaviors. Results: Key risk factors for poor health behaviors included reliance on parental influence (AOR: 5.54; 95% CI: 1.74–17.64) and restricted healthcare access during leisure time (AOR: 4.70; 95% CI: 1.43–15.49). Protective factors included peer influence (AOR: 0.19; 95% CI: 0.05–0.71) and transportation support (AOR: 0.22; 95% CI: 0.06–0.79). Conclusions: Addressing barriers to healthcare access and enhancing social support are critical to promoting healthy behaviors among men in high-risk occupations. Targeted interventions can reduce health disparities and improve outcomes.

## 1. Introduction

Men’s health behaviors are a critical public health issue that significantly influence morbidity, mortality, and healthcare utilization. Globally, men are more likely than women to engage in unhealthy behaviors, such as smoking, excessive alcohol consumption, and poor dietary practices, leading to a higher prevalence of non-communicable diseases (NCDs) and premature mortality [1]. Traditional masculinity norms play a key role in exacerbating these behaviors by discouraging men from seeking preventive healthcare and adopting health-promoting practices [2]. For instance, cultural expectations emphasizing stoicism and self-reliance contribute to delays in seeking medical attention, even for severe health conditions, as observed across diverse global settings [3]. A meta-analysis by Courtenay (2000) found that men who adhere strongly to traditional masculinity norms are less likely to participate in preventive health measures and more likely to engage in risk-taking behaviors [4].

In Malaysia, these global trends are reflected and further shaped by local cultural and occupational factors. The National Health and Morbidity Survey (NHMS) highlights that Malaysian men exhibit lower rates of healthcare utilization and health screenings compared to women, alongside a significantly higher prevalence of smoking (40.5% vs. 1.2%) and risky behaviors such as alcohol consumption (16.9% vs. 6.4%) [5]. Among uniformed service personnel, such as police officers, firefighters, military personnel, and prison staff, these trends are even more concerning due to the demanding nature of their work. Their roles often involve long, irregular working hours, exposure to hazardous environments, and high levels of physical and psychological stress, which contribute to their vulnerability to poor health behaviors [6,7].

Additionally, the educational background of many uniformed service members influences their health behaviors. Entry into these professions in Malaysia generally requires secondary-level education or technical training, which may limit exposure to comprehensive health education programs that promote preventive behaviors [8]. The hierarchical and disciplined nature of these organizations further perpetuates traditional masculinity norms, where health concerns are perceived as a sign of weakness, discouraging individuals from seeking timely medical attention [9]. Occupational stressors within these roles, such as exposure to trauma, heavy workloads, and limited access to mental health resources, often result in unhealthy coping mechanisms, including smoking and alcohol consumption [10,11]. Similar findings have been reported in neighboring countries, such as Singapore and Indonesia, where occupational stress correlates with poor dietary habits and physical inactivity [12].

Despite the growing body of research on men’s health behaviors, there remains limited exploration of these behaviors within the Malaysian context, particularly among public servants in high-stress professions. Moreover, the interaction between occupational stressors, cultural norms, and systemic factors remains poorly understood, underscoring the need for further research to identify specific predictors of health behaviors in this population.

To address these gaps, this study applies Andersen’s Behavioral Model (ABM) as a guiding theoretical framework. ABM categorizes the determinants of health behaviors into predisposing factors (e.g., demographic characteristics, health beliefs), enabling factors (e.g., access to healthcare, socioeconomic status), and need factors (e.g., perceived health status) [13]. The framework provides a comprehensive structure for examining the complex interplay of personal, environmental, and systemic influences on health behaviors. ABM has been widely validated in diverse contexts, making it particularly suitable for studying populations with unique occupational and cultural dynamics, such as Malaysian public servants [14].

This study aims to identify the predictors of health behaviors among male public servants in Kelantan through the lens of ABM. By leveraging the model’s multidimensional approach, this study seeks to generate evidence-based insights that can inform the design of targeted interventions, ultimately improving health outcomes and reducing the burden on Malaysia’s public health system.

## 2. Materials and Methods

### 2.1. Study Design and Setting

This cross-sectional study was conducted between October and December 2023 among male public safety personnel in Kelantan, Malaysia. The study sites included three districts: Kota Bharu, Pasir Mas, and Tanah Merah, which were selected to represent diverse socio-demographic and geographic profiles within Kelantan.

### 2.2. Study Population and Sampling

Participants in this study were male public safety personnel aged 18 to 60 years who were Malaysian citizens and had been actively employed for at least one year. Non-Malaysian personnel were excluded to ensure cultural and systemic consistency in healthcare service utilization. A multistage simple random sampling method was employed to select participants. First, three districts, namely Kota Bharu, Pasir Mas, and Tanah Merah, were randomly chosen to represent diverse socio-demographic and geographic profiles within Kelantan. Within each district, public safety agencies, including the Fire and Rescue Department, the Royal Malaysian Police, and the Civil Defense Force, were identified. Subsequently, individual participants were randomly selected from each agency’s roster using a random number generator. This approach ensured a representative sample of public safety personnel across the selected districts.

This study targeted personnel from uniformed government agencies, including the District Fire and Rescue Office, the District Royal Malaysian Police Office, and the District Malaysia Civil Defense Force Office. These agencies were selected due to their high-demand occupational settings, which often involve irregular schedules, physical strain, and high levels of stress. The nature of work in these agencies makes their employees particularly vulnerable to health risks, providing a unique opportunity to study the determinants of men’s health behaviors.

The sample size was calculated using the G*Power software version 3.1, targeting a medium effect size (0.15) with a power of 80%, a significance level of 0.05, and 30 predictors, yielding a minimum sample size of 187 [15]. Adjusting for a 30% non-response rate, the final target sample size was 267. A total of 257 responses were retained after excluding incomplete questionnaires.

### 2.3. Variables and Instruments

The study utilized Andersen’s Behavioral Model to examine predisposing, enabling, and need factors that influence health behaviors. Predisposing factors included sociodemographics (age, marital status, education, and income), enabling factors covered healthcare access and social support, and need factors included perceived healthcare necessity.

To account for potential confounding effects, sociodemographic factors such as age, marital status, education, and income were included as confounders in the analysis, along with occupational variables such as shift work and job role. These variables were selected based on the prior literature, which highlights their significant influence on men’s health behaviors, particularly in high-risk occupational groups [1,6,8].

Health behaviors were assessed using the Malay Health Behavior Inventory-Short Form (MHBI-SF), a validated 11-item instrument with four subscales: diet, proper use of healthcare resources, anger and stress, and substance use [15]. Responses were rated on the seven-point Likert scale, with higher scores indicating poorer health behaviors. The reliability of the Malay version of the HBI-SF was evaluated using Cronbach’s alpha. Each subscale demonstrated acceptable reliability values: diet (α = 0.763), anger and stress (α = 0.906), proper use of healthcare resources (α = 0.860), and substance use (α = 0.917). These values indicate good internal consistency, supporting the use of this instrument in the current study.

### 2.4. Data Collection

Data collection was conducted in collaboration with district office representatives. Participants were invited to attend scheduled sessions where they received a briefing on the study’s objectives and provided written informed consent. Next, the consent form for participation was distributed to all participants and signed. The questionnaire was self-administered under the supervision of researchers, ensuring confidentiality and addressing participants’ queries. Sensitive topics, such as substance use, were managed with care to minimize response bias.

### 2.5. Statistical Analysis

Data were analyzed using SPSS version 26. Descriptive statistics were used to summarize participant characteristics, presented as frequencies and percentages. Health behavior scores were dichotomized into “good” (scores < 4) and “poor” (scores ≥ 4) based on the total MHBI-SF scores.

Multiple logistic regression was performed to identify factors associated with poor health behaviors, adjusting for potential confounders. Prior to analysis, the normality of continuous variables was assessed using the Kolmogorov–Smirnov test and visual inspection of histograms and Q-Q plots. Variables that were not normally distributed were either categorized or transformed, depending on the nature of the variable, to ensure appropriate analysis. The model fit was evaluated using the Hosmer–Lemeshow test, and interaction terms were checked to rule out multicollinearity. Multicollinearity among predictor variables was assessed using Variance Inflation Factors (VIFs). A threshold of VIF < 10 was used as an indicator of no significant multicollinearity, consistent with recommended practices in regression analysis [16]. The results were reported as adjusted odds ratios (AORs) with 95% confidence intervals (CIs).

## 3. Results

### 3.1. Participant Characteristics

A total of 257 male public safety personnel participated in the study, with a mean age of 40.3 years (SD ± 9.86). Most participants were Malay (99.6%), Muslim (99.6%), and married (83.7%). The majority had secondary education (79.8%), and nearly half (49%) earned a monthly income of MYR 2500–5000. Participants were primarily firefighters (66.9%), followed by civil defense personnel (19.1%) and police officers (14.0%) (Table 1).

### 3.2. Descriptive Analysis of Predisposing, Enabling, and Need Factors

Table 2 presents the descriptive statistics of predisposing, enabling, and need factors influencing healthcare utilization among public safety personnel. Most participants reported no comorbid conditions (83.3%), and a significant majority (84.4%) had sought treatment at public healthcare facilities, though the frequency of visits varied. Social influences, particularly advice from spouses (74.7%) and family members (59.9%), played a crucial role in enabling healthcare utilization, alongside financial and transportation support, which were received by 82.9% and 46.3% of participants, respectively. However, financial constraints (80.5%) and transportation issues (46.7%) remained notable barriers. Awareness initiatives by healthcare providers were also significant, with over half of the participants recognizing the benefits of healthcare and the risks of neglecting it. In terms of need factors, health necessity, such as illness (59.1%) or worsening conditions (56.8%), and self-acknowledged unhealthy lifestyles (45.1%) were major motivators for seeking care. These findings highlight the interplay of systemic barriers, social support, and perceived health needs in shaping healthcare utilization patterns in this population.

### 3.3. Regression Analysis

Table 3 illustrates the findings from the logistic regression analysis. The univariate analysis identified several factors significantly associated with poor health behaviors among public safety personnel. Predisposing factors such as marital status, education level, and income demonstrated associations, while enabling factors, including financial constraints, social support (e.g., advice from spouses, family, and peers), and transportation access, also showed significant relationships. Need factors such as illness, worsening health conditions, and self-acknowledged unhealthy lifestyles were similarly associated. These findings provided the basis for including variables in the multivariate analysis, guided by a *p*-value threshold of <0.25 and clinical relevance [16,17]. Please refer to the Supplementary Materials for detailed results of the simple logistic regression analysis.

Multiple logistic regression analysis identified systemic barriers, such as reliance on parental advice (AOR: 5.54; 95% CI: 1.74–17.64), as significant risk factors. Protective factors included peer influence (AOR: 0.19; 95% CI: 0.05–0.71) and transportation support (AOR: 0.22; 95% CI: 0.06–0.79).

To ensure the validity of the regression model, multicollinearity was assessed using Variance Inflation Factors (VIFs). All predictor variables demonstrated VIF values below the threshold of 10, confirming that no significant multicollinearity was present. The model’s overall fit was validated with a non-significant Hosmer–Lemeshow test (*p* = 0.973), and the classification accuracy was 91.8%.

## 4. Discussion

Health behaviors play a significant role in shaping individual well-being and occupational efficiency, particularly in high-risk professions like public safety. Public safety personnel encounter unique challenges, including irregular schedules and high stress, which shape their health behaviors. This study provides insights into the determinants of these behaviors, emphasizing the need for targeted interventions that address both systemic and social factors to improve healthcare utilization and outcomes.

### 4.1. Systemic Barriers to Healthcare Access

Access to healthcare is a cornerstone of maintaining and improving health outcomes, particularly for individuals in high-stress, high-risk occupations such as public safety personnel. This study identified limited access to healthcare during leisure time as a significant barrier, emphasizing the structural challenges inherent to these roles. Irregular work schedules, including long hours, unpredictable shifts, and emergency responsibilities, often limit opportunities to seek care during conventional operating hours of healthcare facilities. This issue is not merely logistical but carries profound clinical implications [18].

Delayed healthcare access has been consistently linked to poorer health outcomes. Public safety personnel, who are exposed to chronic stress and physically demanding tasks, are at heightened risk for conditions such as cardiovascular disease, musculoskeletal disorders, and mental health issues [19]. Timely access to healthcare is critical for the early diagnosis, management, and prevention of these conditions. For instance, routine screenings for hypertension or diabetes can identify risk factors before they progress to debilitating stages. However, structural barriers, such as restricted access during leisure time, disrupt these preventive efforts, exacerbating chronic conditions and increasing the burden of care [1].

Clinically, the importance of addressing this barrier lies in its potential to improve both individual health outcomes and broader workforce productivity. For public safety personnel, untreated or poorly managed health conditions can impair job performance, increase the likelihood of errors, and elevate risks to public safety. For example, a firefighter with unmanaged hypertension or a police officer with untreated mental health conditions poses risks not only to themselves but also to the communities they serve [20]. Moreover, delayed care often leads to more severe health conditions that require intensive interventions, further straining healthcare systems and increasing costs [21].

Tailored interventions to mitigate this barrier could have substantial clinical and operational benefits. Workplace healthcare services represent a pragmatic solution, bringing preventive and curative services directly to personnel during their shifts. Mobile clinics, on-site health screenings, and telemedicine consultations can address accessibility issues while accommodating irregular schedules. These services have demonstrated success in other high-risk populations, such as military personnel and emergency responders, where similar occupational challenges exist [22]. For instance, studies on workplace health programs in emergency services showed improvements in participation rates for health screenings and adherence to treatment regimens, leading to better health outcomes and reduced absenteeism [23].

Priority access during non-peak hours is another potential intervention. Designating specific time slots or fast-track services for public safety personnel at healthcare facilities could facilitate timely care without disrupting their work responsibilities [24]. However, such interventions require careful coordination between employers and healthcare providers to ensure feasibility and effectiveness. Policymakers and healthcare administrators must allocate resources strategically, ensuring that these tailored services do not compromise access for other populations [17].

Critically, while these solutions address accessibility, their success depends on addressing other underlying factors such as awareness, cultural norms, and logistical barriers. For example, even with workplace healthcare services, personnel may be reluctant to seek care due to stigma or perceived invincibility, a common issue in high-risk professions [19]. Addressing this requires integrated approaches, combining accessibility initiatives with education and cultural shifts that encourage proactive health-seeking behaviors.

The clinical importance of overcoming these barriers extends beyond individual outcomes. Enhanced access to healthcare during leisure time improves workforce readiness, reduces absenteeism, and minimizes the economic and social costs associated with untreated health conditions [21]. Furthermore, by prioritizing the health of public safety personnel, healthcare systems indirectly enhance public safety and community resilience, as these workers are critical to emergency response and crisis management.

### 4.2. Cultural Influences on Health-Seeking Behaviors

The reliance on familial advice reflects deeply ingrained cultural norms, particularly in Malaysian society, where family plays a central role in health-related decision-making. While supportive, this reliance may inadvertently delay professional healthcare engagement when advice lacks medical expertise. From a clinical perspective, this delay can lead to worsening health conditions and missed opportunities for early intervention [21]. Public health strategies that respect cultural dynamics while promoting individual autonomy in healthcare decisions are essential. Educational initiatives focusing on the importance of professional medical advice could improve health-seeking behaviors without undermining familial roles.

### 4.3. The Role of Peer Influence as a Protective Factor

Peer influence emerged as a significant protective factor in this study, underscoring the potential for leveraging workplace camaraderie to foster healthier behaviors among public safety personnel. Team-oriented professions inherently encourage trust and mutual support, creating an environment conducive to positive health interventions. Clinically, this suggests that well-structured, peer-led health promotion programs can improve the adoption and sustainability of health interventions, particularly in high-stress professions.

Peer-led health promotion programs have shown substantial efficacy in fostering health behavior change and mitigating occupational stress. For instance, peer support has been widely utilized in emergency services to address mental health challenges, with studies demonstrating reductions in stress and improvements in resilience and coping mechanisms [19]. By using shared experiences as a foundation, peer-led interventions reduce stigma and encourage openness, leading to the early identification of health concerns and increased engagement with healthcare services [25].

In high-stress occupations such as public safety, structured peer support programs have also been linked to better mental health outcomes. For example, a study on firefighter peer support programs found significant reductions in depressive symptoms and improvements in overall mental well-being when compared to non-participants [26,27]. This aligns with findings in law enforcement, where peer-led initiatives were associated with increased utilization of mental health services, thereby addressing barriers such as stigma and lack of awareness [28].

Peer-led programs function through several mechanisms, including fostering a sense of belonging, providing relatable role models, and enhancing accountability. The trust established within peer groups allows for candid discussions about health concerns, reducing fear of judgment or professional repercussions [1,29]. Moreover, the camaraderie among public safety personnel creates a unique platform for behavioral modeling, where positive health behaviors observed in peers are more likely to be emulated.

From a clinical perspective, the integration of peer support into workplace health promotion offers several advantages. First, it enhances accessibility to mental and physical health resources by embedding these supports within the organizational framework. Second, it empowers personnel to take an active role in their health management, leveraging the credibility and relatability of peers to influence behavior change [29]. Finally, these programs have demonstrated cost-effectiveness, reducing reliance on external healthcare providers and mitigating the long-term costs associated with untreated health conditions [19].

### 4.4. Transportation Support and Logistical Accessibility

Transportation support emerged as a key facilitator of healthcare utilization, emphasizing the importance of logistical accessibility. For public safety personnel, particularly in semi-urban or rural areas, reliable transportation can significantly reduce delays in seeking care. From a clinical perspective, timely healthcare access is crucial for the early detection and management of health conditions [29]. Policies providing subsidized transportation or mobile healthcare units could address these logistical barriers, ensuring equitable healthcare access and improving health outcomes in underserved regions.

### 4.5. Limitations and Future Directions

This study has limitations that warrant consideration. The cross-sectional design prevents causal inference, and the reliance on self-reported data introduces potential bias, particularly in sensitive areas like substance use. Wide confidence intervals (CIs) observed in the regression analysis reflect the sample’s heterogeneity and the relatively small number of participants within certain subgroups. While these findings remain statistically valid, the wide CIs indicate potential variability in the strength of associations and should be interpreted cautiously [30]. Future research should employ larger, more diverse samples to improve the precision of estimates and better capture variability within this population.

Additionally, the study population was limited to public safety personnel in Kelantan, which may restrict generalizability to other regions or occupational groups. Future research should adopt longitudinal designs to assess causality and include diverse geographic and occupational contexts to enhance the robustness of findings. Incorporating qualitative approaches could also provide deeper insights into systemic barriers and coping strategies specific to public safety personnel.

### 4.6. Implications for Research, Practice, and Policy

These findings underscore the importance of tailoring interventions to the occupational realities of public safety personnel.

- Research: Future studies should explore the role of occupational stressors and cultural dynamics in shaping health behaviors among public safety personnel.
- Practice: Healthcare providers should prioritize workplace-based interventions that leverage peer networks and improve logistical access to care. Resilience-building programs integrated into regular training schedules could also enhance coping mechanisms.
- Policy: Policymakers should address structural barriers by providing mobile healthcare units, subsidized transportation, and flexible healthcare services tailored to the unique schedules of public safety personnel.

## 5. Conclusions

This study highlights the critical role of systemic, cultural, and occupational determinants in shaping men’s health behaviors among public safety personnel. Addressing these determinants through tailored interventions and policies has the potential to improve health outcomes, reduce disparities, and promote equity in high-risk occupational settings. By focusing on the unique challenges faced by public safety personnel, this research contributes to the development of targeted strategies that align with their occupational and cultural realities.

## Figures and Tables

**Table 1:** Sociodemographic characteristics of study participants (n = 257).

Variables	Frequency (%)
Age (years)	40.3 (9.86) *
20–29	49 (19.1)
30–39	62 (24.1)
40–49	92 (35.8)
50–59	54 (21.0)
Religion	
Islam	256 (99.6)
Buddhism	1 (0.4)
Ethnicity	
Malay	256 (99.6)
Siamese	1 (0.4)
Status	
Married	215 (83.7)
Single	40 (15.5)
Divorce/widow	2 (0.8)
Educational level	
Primary	1 (0.4)
Secondary	205 (79.8)
Tertiary	51 (19.8)
Occupation	
Fire and Rescue (JBPM)	172 (66.9)
Civil Defence Force (APM)	49 (19.1)
Royal Malaysia Police (PDRM)	36 (14.0)
Monthly individual income (MYR)	
<2500	115 (44.8)
2500–5000	126 (49.0)
5001–7000	11 (4.3)
7001–11,000	5 (1.9)

Indicator: * = mean (SD). Abbreviation: SD = standard deviation.

**Table 2:** Descriptive statistics of the predisposing, enabling, and need factors of men’s healthcare services utilization (n = 257).

Factors	Variables		Frequency (%)
Predisposing	Comorbid	None	214 (83.3)
		Yes	43 (16.7)
	Nearest distance between residence and healthcare facilities (km)		4.1 (3.08) *
	Sought treatment at public healthcare facilities	Ever	217 (84.4)
		Never	40 (15.6)
	Frequency of public healthcare visits	Once monthly	11 (4.3)
		Once quarterly	41 (16.0)
		Twice yearly	71 (27.6)
		One yearly	60 (23.3)
		Biennial/Triennial	34 (13.2)
Enabling	Only obtain advice/persuasion from	Wife/spouse	192 (74.7)
		Family members/relatives	154 (59.9)
		Parents	121 (47.1)
		Friends/colleagues	101 (39.3)
		Neighbor	38 (14.8)
	Only capabilities in terms of	Financial	207 (80.5)
		Leisure time	126 (49.0)
		Transportation	120 (46.7)
	Only provision of aid in terms of	Financial	213 (82.9)
		Transportation	119 (46.3)
	Only unknown health status for a long period	Yes	101 (39.3)
		Unnecessary	99 (38.5)
		No	57 (22.2)
	Only receiving the health screening/treatment benefits explained by healthcare providers	Yes	141 (54.9)
		Unnecessary	71 (27.6)
		No	45 (17.5)
	Only receiving the negative implications of under-optimized health screening/treatment explained by healthcare providers	Yes	145 (56.4)
		Unnecessary	75 (29.2)
		No	37 (14.4)
	Only upon dealing with familiar healthcare providers	Yes	102 (39.7)
		Unnecessary	106 (41.2)
		No	49 (19.1)
Need	Only receiving recommendations made by healthcare providers	Yes	128 (49.8)
		Unnecessary	92 (35.8)
		No	37 (14.4)
	Only suffering due to illness	Yes	152 (59.1)
		Unnecessary	72 (28.0)
		No	33 (12.8)
	Only worsening illness	Yes	146 (56.8)
		Unnecessary	76 (29.6)
		No	35 (13.6)
	Only when self-acknowledge practicing unhealthy lifestyles	Yes	116 (45.1)
		Unnecessary	102 (39.7)
		No	39 (15.2)
	Only believed in strongly needed	Yes	116 (45.1)
		Unnecessary	102 (39.7)
		No	39 (15.2)

Indicator: * = mean (SD). Abbreviation: SD = standard deviation.

**Table 3:** Healthcare services use factors associated with men’s poor health behaviors (n = 257).

Variable		Simple Logistic Regression		Multiple Logistic Regression		
		Crude OR (95% CI)	*p*-Value	B	Adjusted OR (95% CI)	*p*-Value
Sought treatment	Ever	1	0.226	2.190	1	0.06
	Never	3.53 (0.46, 27.20)			8.94 (0.97, 82.53)	
Parents	Not necessary	1	0.151	1.711	1	<0.001
	Yes	2.03 (0.77, 5.33)			5.54 (1.74, 17.64)	
Friends/ colleagues	Not necessary	1		−1.658	1	0.01
	Yes	0.39 (0.13, 1.20)	0.101		0.19 (0.05, 0.71)	
Leisure time	Not necessary	1			1	0.01
	Yes	1.87 (0.71, 4.90)	0.206	1.548	4.70 (1.43, 15.49)	
Provision of transport	Not necessary	1			1	0.02
	Yes	0.51 (0.19, 1.39)	0.188	−1.500	0.22 (0.06, 0.79)	

Constant = −5.315. The backward LR method was applied and reinforced by the enter method. No multicollinearity was detected. No significant interactions among independent variables. The model fits; non-significant Hosmer–Lemeshow test of *p*-value = 0.973, classification table 91.8% correctly classified, and area under ROC curve 77.9% (95% CI: 0.682, 0.875; *p* < 0.001). Abbreviations: OR = odds ratio, CI = confidence interval, B = regression coefficient.

## Data Availability

The data presented in this study are available on request from the corresponding author.

## Supplementary Materials

The following supporting information can be downloaded at https://www.mdpi.com/article/10.3390/healthcare13030291/s1, Supplementary Document: Simple logistic regression of predisposing, enabling, and need factors of healthcare services utilization in relation to poor men’s health behaviors (n = 257).

## Abbreviations

The following abbreviations are used in this manuscript:

MHBI-SF	Malay Health Behavior Inventory-Short Form
AOR	Adjusted Odds Ratios
CI	Confidence Intervals
SD	Standard Deviation
