# Supplementary material for: The Determinants of Men’s Health Behaviors: A Cross-Sectional Study Among Public Safety Personnel in Kelantan, Malaysia
Source: Healthcare (Basel). 2025 Jan 31;13(3):291. doi: 10.3390/healthcare13030291 (PMC11817108; doi:10.3390/healthcare13030291)
Supplement: Supplementary file 1 [file healthcare-13-00291-s001.zip › healthcare-3404156-supplementary.pdf]

Supplementary Document

Simple logistic regression of predisposing, enabling, and need factors of healthcare services utilization in relation to poor men's health behaviors (n = 257).

| **Variable** | **Crude OR (95% CI)** | ***p*-value** |
|---|---|---|
| Age | 1.014 (0.966, 1.065) | 0.565 |
| Distance to healthcare facilities | 1.018 (0.879, 1.179) | 0.809 |
| Educational level | | |
| Tertiary level | 1 | |
| Up to the secondary level | 3.313 (0.429, 25.584) | 0.251 |
| Status | | |
| Single/Divorce | 1 | |
| Married | 1.045 (0.291, 3.759) | 0.946 |
| Occupation | | |
| JBPM | 1 | |
| APM | 1.515 (0.507, 4.531) | 0.457 |
| PDRM | 0.784 (0.168, 3.666) | 0.757 |
| Monthly income (RM) | | |
| 2500 - 11000 | 1 | |
| <2500 | 1.407 (0.552, 3.589) | 0.474 |
| Household income (RM) | | |
| 2500 - >11000 | 1 | |
| <2500 | 1.196 (0.464, 3.085) | 0.711 |
| Comorbid | | |
| No | 1 | |
| Yes | 0.735 (0.232, 2.333) | 0.601 |
| Sought treatment at public healthcare facilities | | |
| Ever | 1 | |
| Never | 3.528 (0.457, 27.203) | 0.226 |
| Advice from Wife/spouse | | |
| Not necessary | 1 | |
| Yes | 0.944 (0.326, 2.730) | 0.915 |
| | | |

| | | |
|---|---|---|
| Advice from Family members/relatives | | |
| Not necessary | 1 | |
| Yes | 0.458 (0.178, 1.182) | 0.106 |
| Advice from Parents | | |
| Not necessary | 1 | |
| Yes | 2.029 (0.772, 5.333) | 0.151 |
| Advice from Friends/colleagues | | |
| Not necessary | 1 | |
| Yes | 0.388 (0.125, 1.203) | 0.101 |
| Financial capabilities | | |
| Not necessary | 1 | |
| Yes | 0.653 (0.224, 1.906) | 0.435 |
| Leisure time | | |
| Not necessary | 1 | |
| Yes | 1.865 (0.710, 4.900) | 0.206 |
| Transportation | | |
| Not necessary | 1 | |
| Yes | 0.510 (0.185, 1.365) | 0.188 |
